# Diversity of words and words for diversity

**DOI:** 10.3389/fpsyt.2026.1834531

**Published:** 2026-06-19

**Authors:** Alexandre Arbey, Caroline Demily, Amelie Soumier

**Affiliations:** 1 Institut de Physique des deux Infinis de Lyon, Université Claude Bernard Lyon 1, Centre National de la Recherche Scientifique (CNRS)/Institut National de Physique Nucléaire et de Physique des Particules (IN2P3), Villeurbanne, France; 2 Genopsy, le Vinatier Hospital Center, Bron cedex, France; 3 Institute of Cognitive Science Marc Jeannerod, Centre National de la Recherche Scientifique Unite Mixte de Recherche (CNRS UMR) 5229 and University of Lyon 1, Bron cedex, France; 4 iMIND, Center of Excellence for Autism, le Vinatier Hospital Center, Bron cedex, France

**Keywords:** autism, community-based-participatory research, neurodiversity, statistics, terminology

## 1 Introduction

In the mid-1960s, Kenneth Burke argued that language acts as a “terministic screen”, like a lens that filters our attention and shapes how we perceive reality (1, 2). The diversity of words, concepts, metaphors, and classificatory schemes categories we rely on, shape how we conceptualize people and interpret their identities, backgrounds, appearances, cultures, and most relevant here, their health-related conditions^3^. In psychiatry, the vocabulary used to describe neurocognitive functioning is far from neutral. It carries implicit value judgments that influence how individuals are treated in clinical, educational, and social spheres. Despite a growing emphasis on neurodiversity-informed practice, a persistent mismatch remains between current clinical language and the lived experiences of neurodivergent stakeholders (1, 3–5). And autism research shifts toward inclusive, community-based participatory approaches (1–11), the need for a shared and nuanced vocabulary, yet scientifically grounded becomes critical. The success of these transdisciplinary research and clinical frameworks depends on a sustained dialogue across distinct perspectives and forms of expertise. In practice, this dialogue can be productive if participants share a vocabulary that is explicit, carefully defined, and sufficiently nuanced to travel across contexts, community settings, scientific disciplines, clinical care, education and policy. Without such shared language, the same terms can be perceived differently, limiting mutual understanding. It is precisely in this context that the present article has emerged. This Opinion article, born from a collaboration between an autistic astrophysicist, a neuroscientist, and a clinician, argues that terminological precision is a prerequisite for meaningful advancement of true inclusive research.

## 2 The power of terminology: beyond deficit-based framing

Our working group discussions, alongside published reviews and guidelines, underscore a straightforward point: autism-related terminology is not neutral. The past decades have brought a major shift in how autism is understood in relation to identity. The words used in research, clinical practice, media and public discourse can either empower and support autistic people, and reduce stigma, or conversely sustain medicalized, pathologizing and deficit-based approaches (8). Terminology does more than reflect scientific findings, as it structures research questions, shapes funding priorities, and influences the clustering of human traits. Historically, autism-related language has leaned toward medicalized and pathologizing models. While there is no universal consensus on labels (12), such as “autistic person” versus “person with autism”, the shift toward identity-affirming language is a vital step in reducing stigma and in empowering the community (12–15). Therefore, terminological precision should not be solely viewed as an abstract philosophical exercise but as a practical necessity for ensuring that dialogue between researchers, practitioners, and neurodivergent stakeholders remains both meaningful and inclusive.

The term “neurodiversity”, a concept that has rapidly circulated, often with divergent definitions and normative implications (16, 17), illustrates this shift. It refers to neurological diversity, e.g. the natural variation in human neurocognitive functioning (18), and reframes neurological variation as a natural biological reality rather than a series of “abnormalities”. The term is used to encompasses a broad range of genetic, neurological, psychological, behavioral and cognitive differences, encouraging a re-examination of fixed oppositions such as “normal/typical” versus “abnormal/atypical” (19, 20). However, the inconsistent application of terms like “neuroatypicality” or “neurodivergence” often fuels misunderstanding, and even the term « neurodivergence » remains contested and debated (21–24). Clarity in these distinctions (see **Supplementary File**) is essential for practitioners to distinguish between a constellation of traits and the functional distress those traits may cause in unadapted environments. For instance, while “disorder” implies clinically significant impairment within a specific environment, “spectrum” should be understood as a continuum of human variation. From a medical perspective, identifying similarities is critical for developing remedies and interventions that are applicable to the majority (25–28). This requires the definition of a standard, a norm or a reference point (29, 30). While physiological differences can be easily identifiable, establishing clear boundaries between “normal” or “typical and “abnormal” or ‘atypical” human neurocognitive functioning and behavior proves complex (31). However, extending this framework to heterogeneous neurodevelopmental conditions such as autism raises important difficulties. In autism, appeals to a single “norm” can obscure substantial variation in presentation, developmental trajectories, and underlying mechanisms, and may therefore oversimplify what is best understood as a spectrum of profiles (32–34).

Despite the increasing adoption of neurodiversity-affirming language, the terminology used to characterize neurocognitive functioning remains a subject of intense public and scientific debate. We believe that this persistent lack of consensus is largely driven by the fact that traditional norm-referenced statistical approaches, which define “normality” or “typicality” may not be fully suited to analyse the complex patterns of trait distribution, clustering, and co-occurrence inherent to the spectrum-based nature of neurodevelopmental conditions (21, 32, 33, 35, 36).

## 3 The limits of statistical norms in neurodivergent assessment

A significant challenge in current practice is the heavy reliance on norm-referenced statistical approaches. In many statistical settings, “typical” functioning is defined as falling within the central range of a reference population, which is approximated within ±2 standard deviations of the mean (37, 38). This presupposes that robust, representative normative datasets exist for meaningful comparisons between groups (39, 40). While this works for biomarkers like blood pressure or glucose level, it is less reliable for neurocognitive functioning, where assessment often depends on behavioral observation and standardized scales, with limited cohorts and sample sizes. Many biological variables of interest (e.g. neurotransmitter dynamics) cannot be easily measured in the living brain, and clinical assessment therefore relies on behavioral observation and informant-rated scales (41–43). Accordingly, neurodevelopmental conditions, including ASD, ADHD, and specific learning disabilities (dyslexia, dysgraphia, dyscalculia), are commonly identified through clinicians’ interpretation of standardized tests and questionnaires (44–46).

Standard Intelligent Quotient (IQ) tests, that are widely used in clinical assessment and sometimes in selection contexts such as education or employment, are designed to measure performance relative to a neurotypical reference norm and samples. Atypical reasoning styles may lead to “incorrect” answers on these tests not because of diminished ability, but because the individual utilizes an alternative, defensible inferential path that the test’s conventions do not account for. Series-completion items can admit multiple valid continuations in the absence of an explicit generative principle, and verbal reasoning tasks can be sensitive to linguistic and cultural differences. In such cases, very high or very low scores may be difficult to interpret as straightforward indices of “intelligence.” Rather, they may partly index conformity with a particular style of problem framing and normative logic (47, 48).

Consequently, IQ scores can underestimate the true cognitive potential of neurodivergent individuals, reflecting a “test mismatch” rather than inherent deficiency (47–49). This reliance on population-based benchmarks is further illustrated by the category of ‘High Intellectual Potential’, which utilizes a threshold of two standard deviations above the mean to sort individuals into discrete labels based on statistical distribution rather than functional reality. Together, these examples suggest that traditional psychometrics may often measure an individual’s conformity to a specific normative logic and problem-framing style rather than providing an objective assessment of inherent neurocognitive capacity. In other words, low or high IQ does not inherently imply neurodivergence or disorder; instead, neurodivergence can substantially influence how a person performs on IQ tasks (49–51, 74). This underscores the need for assessment approaches that are sensitive to different cognitive profiles and that distinguish between “test fit” and inherent underlying abilities (21, 52, 53). This immediately raises questions about where thresholds should be placed, what they capture (or miss) about real-world functioning, and how context shapes whether a difference becomes a disability (36, 40, 54–56).

Furthermore, using statistical cut-offs to define “disorder” is problematic when considering prevalence. ADHD, estimated at around 10% worldwide, on the order of one billion people, has a prevalence comparable to diabetes (10.5%) (57, 58), and often falls within the range of typical variation statistically (less than two standard deviations of the mean), whereas autism [approx. 1% (59)] is statistically “atypical”. However, ADHD and autism frequently co-occur, and a substantial subset of autistic individuals also meet criteria for ADHD (and vice versa) (60, 61). Drawing a hard line between variation and disorder based solely on thresholds fails to capture the complexity of co-occurring traits and real-world functioning (36, 52, 54, 55).

## 4 Discussion

Establishing a shared language for neurodivergence and neurodiversity remains a persistent challenge, as inconsistent terminology continues to fuel misunderstanding and erode trust within the community (12, 15–17, 20). Because a substantial portion of the population experiences neurodevelopmental differences, the neurodiversity framework must challenge inherited assumptions of “normality” and standardization. This shift moves the scientific discussion beyond mere statistics toward broader questions of social participation and how society manages cognitive difference (62). Historically, atypical cognitive profiles have been relegated to varied roles, from inventors to social outcasts, yet societies have repeatedly organized these differences through a logic of “boundary-making” that dictates which individuals are considered “legitimate” (63–67). These dynamics persist in contemporary discourse; while there is growing recognition of neurodiversity as ordinary human variation, stereotypes such as being “super smart” or “prone to outbursts” remain prevalent. Furthermore, well-intentioned but trivializing remarks, such as “everyone is a little autistic,” can unintentionally diminish the significant challenges faced by autistic individuals. The limitations of a purely deficit-based lens can be illustrated through the analogy of an invented “Old Age Spectrum Disorder”. While aging is a universal and highly variable experience, pathologizing its associated difficulties under a single spectrum label would likely encourage overgeneralization and promote “one-size-fits-all” interventions. Such a label would also stigmatize those who do not identify with the diagnostic criteria and distort research outcomes; studies recruiting only “diagnosed” individuals would fail to capture the broader continuum of the aging experience. Similarly, in neurodiversity research, a narrow focus on diagnosed groups introduces systematic bias into how we define, measure, and interpret neurodevelopmental diversity (68, 69).

Beyond a strictly medical framework, autistic language may also be read through an interpretive lens. From this perspective, language is not reduced linguistic markers alone, but is understood as rhetorically patterned, semantically layered and interpretive plurality. This perspective resonates with practical criticism and theories of ambiguity, which emphasize the role of formal features, contextual nuance, and the capacity of language to sustain several meanings at once (70–72).

To conclude, neurodevelopmental diversity should be recognized as a meaningful source of creativity, insight, and adaptability. Valuing diverse ways of thinking, and engaging neurodivergent people as genuine partners in community-based participatory research can strengthen scientific rigor, and ensures findings remain relevant to the population being studied (17, 69, 73). Reframing neurodevelopmental conditions within a broader understanding of human diversity does not deny the reality of significant difficulties or the need for clinical support. Instead, it invites practitioners and researchers to attend simultaneously to an individual’s strengths, needs, and environmental context.

## References

[1] Keating CT Hickman L Leung J Monk R Montgomery A Heath H . Autism-related language preferences of English-speaking individuals across the globe: A mixed methods investigation. Autism Res. (2023) 16:406–28. doi: 10.31234/osf.io/859x3 36474364 PMC10946540

[2] Ostaszewska A Harper G Davis R Joseph H . Beyond diagnosis: Setting research priorities with the neurodivergent community. Neurodiversity. (2025) 3:27546330251374236. doi: 10.1177/27546330251374236

[3] Den Houting J Higgins J Isaacs K Mahony J Pellicano E . ‘I’m not just a Guinea pig’: Academic and community perceptions of participatory autism research. Autism. (2021) 25:148–63. doi: 10.1177/1362361320951696 32854511

[4] Fletcher-Watson S Brook K Hallett S Murray F Crompton CJ . Inclusive practices for neurodevelopmental research. Curr Dev Disord Rep. (2021) 8:88–97. doi: 10.1007/s40474-021-00227-z 30311153

[5] Fletcher-Watson S Adams J Brook K Charman T Crane L Cusack J . Making the future together: Shaping autism research through meaningful participation. Autism. (2019) 23:943–53. doi: 10.1177/1362361318786721 30095277 PMC6512245

[6] Den Houting J Higgins J Isaacs K Mahony J Pellicano E . From ivory tower to inclusion: Stakeholders’ experiences of community engagement in Australian autism research. Front Psychol. (2022) 13:876990. doi: 10.3389/fpsyg.2022.876990 36092113 PMC9454607

[7] Pickard H Pellicano E Den Houting J Crane L . Participatory autism research: Early career and established researchers’ views and experiences. Autism. (2022) 26:75–87. doi: 10.1177/13623613211019594 34088215 PMC8750139

[8] Keating CT . Participatory autism research: How consultation benefits everyone. Front Psychol. (2021) 12:713982. doi: 10.3389/fpsyg.2021.713982 34504463 PMC8421540

[9] Pieron M Lacroix A Chokron S Demily C . Pour une recherche participative dans le trouble du spectre de l’autisme. L’Encéphale. (2025) 51:563–5. doi: 10.1016/j.encep.2024.11.020 39956666

[10] Nicolaidis C Raymaker D Kapp SK Baggs A Ashkenazy E McDonald K . The AASPIRE practice-based guidelines for the inclusion of autistic adults in research as co-researchers and study participants. Autism. (2019) 23:2007–19. doi: 10.1177/1362361319830523 30939892 PMC6776684

[11] Hobson H Linden A Crane L Kalandadze T . Towards reproducible and respectful autism research: Combining open and participatory autism research practices. Res Autism Spectr Disord. (2023) 106:102196. doi: 10.31234/osf.io/zdns5 42059414

[12] Kenny L Hattersley C Molins B Buckley C Povey C Pellicano E . Which terms should be used to describe autism? Perspectives from the UK autism community. Autism. (2016) 20:442–62. doi: 10.1177/1362361315588200 26134030

[13] Buijsman R Begeer S Scheeren AM . ‘Autistic person’ or ‘person with autism’? Person-first language preference in Dutch adults with autism and parents. Autism. (2023) 27:788–95. doi: 10.1177/13623613221117914 PMC10074744 35957517

[14] Monk R Whitehouse AJO Waddington H . The use of language in autism research. Trends Neurosci. (2022) 45:791–3. doi: 10.1016/j.tins.2022.08.009 36184384

[15] Taboas A Doepke K Zimmerman C . Preferences for identity-first versus person-first language in a US sample of autism stakeholders. Autism. (2023) 27:565–70. doi: 10.1177/13623613221130845 36237135

[16] Bottema-Beutel K Kapp SK Lester JN Sasson NJ Hand BN . Avoiding ableist language: Suggestions for autism researchers. Autism Adulthood. (2021) 3:18–29. doi: 10.1089/aut.2020.0014 36601265 PMC8992888

[17] Dwyer P . The neurodiversity approach(es): What are they and what do they mean for researchers? Hum Dev. (2022) 66:73–92. doi: 10.1159/000523723 36158596 PMC9261839

[18] Botha M Hanlon J Williams GL . Does language matter? Identity-first versus person-first language use in autism research: A response to Vivanti. J Autism Dev Disord. (2023) 53:870–8. doi: 10.1007/s10803-020-04858-w 33474662 PMC7817071

[19] Rosqvist HB Chown N Stenning A eds. Neurodiversity studies: A new critical paradigm. New York, NY, United States: Routledge Taylor & Francis Group (2020).

[20] Sonuga-Barke E Thapar A . The neurodiversity concept: Is it helpful for clinicians and scientists? Lancet Psychiatry. (2021) 8:559–61. doi: 10.1016/s2215-0366(21)00167-x 33984295

[21] Apperly IA Lee R Van Der Kleij SW Devine RT . A transdiagnostic approach to neurodiversity in a representative population sample: The N+ 4 model. JCPP Adv. (2024) 4:e12219. doi: 10.1002/jcv2.12219 38827989 PMC11143952

[22] Parenti I Rabaneda LG Schoen H Novarino G . Neurodevelopmental disorders: From genetics to functional pathways. Trends Neurosci. (2020) 43:608–21. doi: 10.1016/j.tins.2020.05.004 32507511

[23] Cainelli E Bisiacchi P . Neurodevelopmental disorders: Past, present, and future. Children. (2022) 10:31. doi: 10.3390/children10010031 36670582 PMC9856894

[24] Goldberg H . Unraveling neurodiversity: Insights from neuroscientific perspectives. Encyclopedia. (2023) 3:972–80. doi: 10.3390/encyclopedia3030070 30654563

[25] Vogel M Ebert C Gensichen J Applis H Hasan A Lochbühler K . A systematic review and meta-analysis of transdiagnostic interventions for common mental disorders in primary care. Gen Hosp Psychiatry. (2024) 91:167–79. doi: 10.1016/j.genhosppsych.2024.11.003 39557003

[26] Insel T Cuthbert B Garvey M Heinssen R Pine DS Quinn K . Research domain criteria (RDoC): Toward a new classification framework for research on mental disorders. AJP. (2010) 167:748–51. doi: 10.1176/appi.ajp.2010.09091379 20595427

[27] Holmes EA Ghaderi A Harmer CJ Ramchandani PG Cuijpers P Morrison AP . The Lancet Psychiatry Commission on psychological treatments research in tomorrow’s science. Lancet Psychiatry. (2018) 5:237–86. doi: 10.1016/s2215-0366(17)30513-8 29482764

[28] Gallagher MW . Transdiagnostic mechanisms of change and cognitive-behavioral treatments for PTSD. Curr Opin Psychol. (2017) 14:90–8. doi: 10.1016/j.copsyc.2016.12.002 28813326

[29] Cohen JF Korevaar DA Altman DG Bruns DE Gatsonis CA Hooft L . STARD 2015 guidelines for reporting diagnostic accuracy studies: Explanation and elaboration. BMJ Open. (2016) 6:e012799. doi: 10.1136/bmjopen-2016-012799 28137831 PMC5128957

[30] Pauker SG Kassirer JP . The threshold approach to clinical decision making. N Engl J Med. (1980) 302:1109–17. doi: 10.1056/nejm198005153022003 7366635

[31] Chiang H-M Tsai LY Cheung YK Brown A Li H . A meta-analysis of differences in IQ profiles between individuals with Asperger’s disorder and high-functioning autism. J Autism Dev Disord. (2014) 44:1577–96. doi: 10.1007/s10803-013-2025-2 24362849

[32] Happé F Frith U . Annual research review: Looking back to look forward – changes in the concept of autism and implications for future research. Child Psychol Psychiatry. (2020) 61:218–32. doi: 10.1111/jcpp.13176 31994188

[33] Lombardo MV Lai M-C Baron-Cohen S . Big data approaches to decomposing heterogeneity across the autism spectrum. Mol Psychiatry. (2019) 24:1435–50. doi: 10.1038/s41380-018-0321-0 30617272 PMC6754748

[34] Lord C Bishop S Anderson D . Developmental trajectories as autism phenotypes. Am J Med Genet Pt C. (2015) 169:198–208. doi: 10.1002/ajmg.c.31440 25959391 PMC4898819

[35] Vargason T Frye RE McGuinness DL Hahn J . Clustering of co‐occurring conditions in autism spectrum disorder during early childhood: A retrospective analysis of medical claims data. Autism Res. (2019) 12:1272–85. doi: 10.1002/aur.2128 31149786 PMC6688922

[36] Farmer C Thurm A Troy JD Kaat AJ . Comparing ability and norm-referenced scores as clinical trial outcomes for neurodevelopmental disabilities: A simulation study. J Neurodevelop Disord. (2023) 15:4. doi: 10.31234/osf.io/p4vjx 36650450 PMC9843928

[37] Martinez-Sanchez L Marques-Garcia F Ozarda Y Blanco A Brouwer N Canalias F . Big data and reference intervals: Rationale, current practices, harmonization and standardization prerequisites and future perspectives of indirect determination of reference intervals using routine data. Adv Lab Med / Avances En Med Laboratorio. (2021) 2:9–16. doi: 10.1515/almed-2020-0034 37359198 PMC10197285

[38] Yang D Su Z Zhao M . Big data and reference intervals. Clin Chim Acta. (2022) 527:23–32. doi: 10.1016/j.cca.2022.01.001 34999059

[39] Constantino JN Davis SA Todd RD Schindler MK Gross MM Brophy SL . Validation of a brief quantitative measure of autistic traits: Comparison of the Social Responsiveness Scale with the Autism Diagnostic Interview-Revised. J Autism Dev Disord. (2003) 33:427–33. doi: 10.1023/a:1025014929212 12959421

[40] Gotham K Pickles A Lord C . Standardizing ADOS scores for a measure of severity in autism spectrum disorders. J Autism Dev Disord. (2009) 39:693–705. doi: 10.1007/s10803-008-0674-3 19082876 PMC2922918

[41] Jones RM Lord C . Diagnosing autism in neurobiological research studies. Behav Brain Res. (2013) 251:113–24. doi: 10.1016/j.bbr.2012.10.037 23153932 PMC3711973

[42] Ceccarini J Liu H Van Laere K Morris ED Sander CY . Methods for quantifying neurotransmitter dynamics in the living brain with PET imaging. Front Physiol. (2020) 11:792. doi: 10.3389/fphys.2020.00792 32792972 PMC7385290

[43] Paterson LM Tyacke RJ Nutt DJ Knudsen GM . Measuring endogenous 5-HT release by emission tomography: Promises and pitfalls. J Cereb Blood Flow Metab. (2010) 30:1682–706. doi: 10.1038/jcbfm.2010.104 20664611 PMC3023404

[44] Adamou M Arif M Asherson P Cubbin S Leaver L Sedgwick-Müller J . The adult ADHD assessment quality assurance standard. Front Psychiatry. (2024) 15:1380410. doi: 10.3389/fpsyt.2024.1380410 39156609 PMC11327143

[45] Curnow E Utley I Rutherford M Johnston L Maciver D . Diagnostic assessment of autism in adults – current considerations in neurodevelopmentally informed professional learning with reference to ADOS-2. Front Psychiatry. (2023) 14:1258204. doi: 10.3389/fpsyt.2023.1258204 37867776 PMC10585137

[46] Holden C Kirby P Snowling MJ Thompson PA Carroll JM . Towards a consensus for dyslexia practice: Findings of a Delphi study on assessment and identification. Dyslexia. (2025) 31:e1800. doi: 10.35542/osf.io/g7m8n 39996532 PMC11852196

[47] Kirby JR Lawson MJ . Effects of strategy training on Progressive Matrices performance. Contemp Educ Psychol. (1983) 8:127–40. doi: 10.1016/0361-476x(83)90004-8

[48] Kunda M Soulières I Rozga A Goel AK . Error patterns on the raven’s standard progressive matrices test. Intelligence. (2016) 59:181–98. doi: 10.1016/j.intell.2016.09.004 38826717

[49] Grondhuis SN Lecavalier L Arnold LE Handen BL Scahill L McDougle CJ . Differences in verbal and nonverbal IQ test scores in children with autism spectrum disorder. Res Autism Spectr Disord. (2018) 49:47–55. doi: 10.1016/j.rasd.2018.02.001 38826717

[50] Goodwin E Gudjonsson GH Sigurdsson JF Young S . The impact of ADHD symptoms on intelligence test achievement and speed of performance. Pers Individ Dif. (2011) 50:1273–7. doi: 10.1016/j.paid.2011.02.023 38826717

[51] Dennis M Francis DJ Cirino PT Schachar R Barnes MA Fletcher JM . Why IQ is not a covariate in cognitive studies of neurodevelopmental disorders. J Int Neuropsychol Soc. (2009) 15:331–43. doi: 10.1017/s1355617709090481 19402919 PMC3075072

[52] Mottron L Bzdok D . Autism spectrum heterogeneity: fact or artifact? Mol Psychiatry. (2020) 25:3178–85. doi: 10.1038/s41380-020-0748-y 32355335 PMC7714694

[53] Klin A . Biomarkers in autism spectrum disorder: challenges, advances, and the need for biomarkers of relevance to public health. FOC. (2018) 16:135–42. doi: 10.1176/appi.focus.20170047 31975908 PMC6526849

[54] Robinson EB Munir K Munafò MR Hughes M McCormick MC Koenen KC . Stability of autistic traits in the general population: further evidence for a continuum of impairment. J Am Acad Child Adolesc Psychiatry. (2011) 50:376–84. doi: 10.1016/j.jaac.2011.01.005 21421177 PMC3174769

[55] Üstün TB Chatterji S Bickenbach J Kostanjsek N Schneider M . The International Classification of Functioning, Disability and Health: a new tool for understanding disability and health. Disability Rehabil. (2003) 25:565–71. doi: 10.1080/0963828031000137063 12959329

[56] Nguyen PH Ocansey ME Miller M Le DTK Schmidt RJ Prado EL . The reliability and validity of the social responsiveness scale to measure autism symptomology in Vietnamese children. Autism Res. (2019) 12:1706–18. doi: 10.1002/aur.2179 31355545 PMC7397486

[57] Ayano G Demelash S Gizachew Y Tsegay L Alati R . The global prevalence of attention deficit hyperactivity disorder in children and adolescents: an umbrella review of meta-analyses. J Affect Disord. (2023) 339:860–6. doi: 10.1016/j.jad.2023.07.071 37495084

[58] Sun H Saeedi P Karuranga S Pinkepank M Ogurtsova K Duncan BB . IDF Diabetes Atlas: global, regional and country-level diabetes prevalence estimates for 2021 and projections for 2045. Diabetes Res Clin Pract. (2022) 183:109119. doi: 10.1016/j.diabres.2021.109119 34879977 PMC11057359

[59] Zeidan J Fombonne E Scorah J Ibrahim A Durkin MS Saxena S . Global prevalence of autism: a systematic review update. Autism Res. (2022) 15:778–90. doi: 10.1002/aur.2696 35238171 PMC9310578

[60] Polderman TJC Hoekstra RA Posthuma D Larsson H . The co-occurrence of autistic and ADHD dimensions in adults: an etiological study in 17 770 twins. Transl Psychiatry. (2014) 4:e435–5. doi: 10.1038/tp.2014.84 25180574 PMC4203013

[61] Rong Y Yang C-J Jin Y Wang Y . Prevalence of attention-deficit/hyperactivity disorder in individuals with autism spectrum disorder: a meta-analysis. Res Autism Spectr Disord. (2021) 83:101759. doi: 10.1016/j.rasd.2021.101759 38826717

[62] Kirkbride JB Anglin DM Colman I Dykxhoorn J Jones PB Patalay P . The social determinants of mental health and disorder: evidence, prevention and recommendations. World Psychiatry. (2024) 23:58–90. doi: 10.1002/wps.21160 38214615 PMC10786006

[63] Chiri G Bergey M Mackie TI . Deserving but not entitled: the social construction of autism spectrum disorder in federal policy. Soc Sci Med. (2022) 301:114974. doi: 10.1016/j.socscimed.2022.114974 35452891

[64] Han E Scior K Avramides K Crane L . A systematic review on autistic people’s experiences of stigma and coping strategies. Autism Res. (2022) 15:12–26. doi: 10.1002/aur.2652 34881514

[65] Hungerford C Kornhaber R West S Cleary M . Autism, stereotypes, and stigma: the impact of media representations. Issues Ment Health Nurs. (2025) 46:254–60. doi: 10.1080/01612840.2025.2456698 39913900

[66] John RP Knott FJ Harvey KN . Myths about autism: an exploratory study using focus groups. Autism. (2018) 22:845–54. doi: 10.1177/1362361317714990 28778133

[67] Link BG Phelan JC . Conceptualizing stigma. Annu Rev Sociol. (2001) 27:363–85. doi: 10.1146/annurev.soc.27.1.363 41139587

[68] LeFevre-Levy R Melson-Silimon A Harmata R Hulett AL Carter NT . Neurodiversity in the workplace: considering neuroatypicality as a form of diversity. Ind Organ Psychol. (2023) 16:1–19. doi: 10.1017/iop.2022.86 41292463

[69] Schuck RK Tagavi DM Baiden KMP Dwyer P Williams ZJ Osuna A . Neurodiversity and autism intervention: reconciling perspectives through a naturalistic developmental behavioral intervention framework. J Autism Dev Disord. (2022) 52:4625–45. doi: 10.1007/s10803-021-05316-x 34643863 PMC9508016

[70] Empson W . Seven types of ambiguity. London: Hogarth (1991).

[71] Richards IA . Practical criticism: a study of literary judgment. San Diego [Calif.] New York London: Harcourt Brace Jovanovich (1980).

[72] Burke K . A grammar of motives. Berkeley, CA: University of California Press (2020).

[73] Armstrong T . The myth of the normal brain: embracing neurodiversity. AMA J Ethics. (2015) 17:348–52. doi: 10.1001/journalofethics.2015.17.4.msoc1-1504 25901703

[74] Courchesne V Bedford R Pickles A Duku E Kerns C Mirenda P . Non-verbal IQ and change in restricted and repetitive behavior throughout childhood in autism: A longitudinal study using the Autism Diagnostic Interview-Revised. Mol Autism. (2021) 12:57. doi: 10.1186/s13229-021-00461-7 34391468 PMC8364071

