# Supplementary material for: Diversity of words and words for diversity
Source: Front Psychiatry. 2026 Jun 19;17:1834531. doi: 10.3389/fpsyt.2026.1834531 (PMC13328447; doi:10.3389/fpsyt.2026.1834531)
Supplement: Supplementary file 1 [file SupplementaryFile1.docx]

Supplementary File 1

Title

Diversity of words and words of diversity

**Description**

This glossary clarifies the nuanced terminology commonly used in the autism and neurodiversity field, including “neurodiversity,” “neurodivergence,” “neuroatypicality,” “spectrum,” and related clinical terms. By exploring the etymological roots, definitions, and connotations of each term, it highlights subtle distinctions that are often overlooked in everyday and scientific discourse. Notably, this glossary presents an original perspective grounded in the semantic reflection of a neurodivergent scientist, offering insight that is rarely captured in academic literature.

**Glossary**

Many terms are used interchangeably in the field of neurodiversity, often with overlapping meanings. However, their etymological roots reveal subtle distinctions that, when acknowledged, can help toward greater inclusion.

**Neurodiversity** (adj.: **neurodiverse**): refers to the natural range of differences in individual neurological and psychological functioning within the human population reflecting what is considered “normal” variations. As a term, neurodiversity encompasses the majority of the human population. It emphasizes inclusion*,* particularly by highlighting the continuous “spectra” of behaviors. The adjective “neurodiverse”, “in the direction of neurodiversity”, primarily relates to actions or initiatives that support or promote neurodiversity, rather than being applicable to individuals.

**Neurodivergence** (adj.: **neurodivergent**): refers to divergence in neurological or psychological functioning that deviates from what is considered normal or typical. However, the definition of this term is inherently ambiguous, as the concepts of “typical” or “normal” are contextual and variable, as they are strongly influenced by social, societal and familial contexts, as well as prevailing prejudices. This fluidity in definition reflects the complex nature of neurodiversity and the challenges in establishing fixed categories in human neurocognitive functioning. The adjective “neurodivergent” should be used to describe individuals with non-standard behavioral or mental functioning. It is often applied to those with conditions such as ASD or ADHD, frequently carrying negative connotations. Paradoxically, it’s also used to describe individuals with exceptionally high IQ scores, but in these cases with a positive connotation. Due to their contextual definitions and associated connotations, the terms "neurodivergence" and "neurodivergent" should be used cautiously in discussions on neurodiversity. Their use is more appropriate when referring to clear neurobiological differences that are distinctly separate from the typical human neurobiological architecture, rather than for describing variations along a continuous spectrum of human neurocognitive functioning.

**Neuroatypicality** (adj.: **neuroatypical**): refers to non-standard neurological or psychological functioning. While its definition appears similar to “neurodivergence”, the connotations and usage of these terms differ significantly. In everyday language, “atypical” is commonly used to describe the broad variability of human traits without inherent a priori positive or negative judgments. This neutral connotation makes “neuroatypical” a more inclusive term compared to “neurodivergent” Given its more neutral and inclusive nature, "neuroatypical" is generally preferred over "neurodivergent" in discussions.

**Spectrum**: the term “spectrum,” originally from physics, has been adopted in psychiatry to describe a range of related conditions. In this context, a spectrum refers to a group of conditions that appear qualitatively distinct but are believed to share underlying neurological connections.

**Autistic spectrum**: the autistic spectrum encompasses a continuum of autistic characteristic traits. Following the general idea that “everyone has autistic features” of varying degrees, the concept of “autistic spectrum” posits a seamless progression from individuals exhibiting minimal autistic traits to those displaying pronounced autistic characteristics. As a consequence, the autistic spectrum refers to a group of traits, highlighting the natural variability within the human diversity.

**Mental/psychic/neurological disorder**: is a clinically significant disruption in neurological or psychological functioning, often synonymous with “disease”, “syndrome” or “condition”. It can be acquired like depression, or congenital, like intellectual disability, impacting cognition, emotion, behavior, and overall function

**Neurodevelopmental disorders (NDD)**: are neurological conditions originating before birth, affecting both brain development and function. While autism is often classified as an NDD, its conceptualization as a spectrum challenges this categorization. The autism spectrum represents a diverse range of traits rather than a specific disorder.

**Autism Spectrum Disorder (ASD)**: even if often used as a synonym of “autism”, ASD refers to the mental/psychic disorders which are generally associated with “autism”. These disorders are the consequences of the expression of autistic traits in a given and not-adapted environment, meaning that an individual with strong autistic traits does not necessarily has an ASD, while an individual with ASD has clear autistic traits. In other words, it is important to distinguish “autism” (a group of traits in the human neurodiversity) from “ASD” (set of mental/psychic disorders which impair an individual with clear autistic traits). Thus, autist individuals can be neuroatypical without being neurodivergent, whereas individuals with ASD can be seen as neurodivergent in the sense that there are impaired in their standard environment.

**Attention Deficit Hyperactivity Disorder (ADHD)**: hyperactivity, impulsivity and attentiveness are individual traits, and similarly to autism, they are not necessarily related to disorders. Since about 10% of individuals are estimated to have ADHD, people with ADHD cannot, except for extreme cases, be considered as neuroatypical or neurodivergent, because they are a non-negligible minority which is not far away from the “norm”. It is important to remember that *not* been neurodivergent/neuroatypical does not mean *not* having a disorder.
